# Single-strand DNA library preparation improves sequencing of formalin-fixed and paraffin-embedded (FFPE) cancer DNA

**DOI:** 10.18632/oncotarget.10827

**Published:** 2016-07-24

**Authors:** Mathias Stiller, Antje Sucker, Klaus Griewank, Daniela Aust, Gustavo Bruno Baretton, Dirk Schadendorf, Susanne Horn

**Affiliations:** ^1^ Department of Evolutionary Genetics, Max Planck Institute for Evolutionary Anthropology, D-04103 Leipzig, Germany; ^2^ Department of Dermatology, University Hospital, West German Cancer Center, University Duisburg-Essen, and German Consortium for Translational Cancer Research (DKTK), D-45147 Essen, Germany; ^3^ Department for Translational Skin Cancer Research, University Duisburg-Essen, and German Consortium for Translational Cancer Research (DKTK), D-45141 Essen, Germany; ^4^ Departments of Surgery and Pathology, Technical University Dresden, D-01307 Dresden, Germany

**Keywords:** high-throughput sequencing, library preparation, formalin-fixed paraffin embedded (FFPE) tissue, cancer, whole exome sequencing

## Abstract

DNA derived from formalin-fixed and paraffin-embedded (FFPE) tissue has been a challenge to large-scale genomic sequencing, due to its low quality and quantities. Improved techniques enabling the genome-wide analysis of FFPE material would be of great value, both from a research and clinical perspective.

Comparing a single-strand DNA library preparation method originally developed for ancient DNA to conventional protocols using double-stranded DNA derived from FFPE material we obtain on average 900-fold more library molecules and improved sequence complexity from as little as 5 ng input DNA. FFPE DNA is highly fragmented, usually below 100bp, and up to 60% of reads start after or end prior to adenine residues, suggesting that crosslinks predominate at adenine residues. Similar to ancient DNA, C > T substitutions are slightly increased with maximum rates up to 3% at the ends of molecules. In whole exome sequencing of single-strand libraries from lung, breast, colorectal, prostate and skin cancers we identify known cancer mutations. In summary, we show that single-strand library preparation enables genomic sequencing, even from low amounts of degraded FFPE DNA. This method provides a clear advantage both in research and clinical settings, where FFPE material (e.g. from biopsies) often is the only source of DNA available. Improving the genetic characterization that can be performed on conventional archived FFPE tissue, the single-strand library preparation allows scarce samples to be used in personalized medicine and enables larger sample sizes in future sequencing studies.

## INTRODUCTION

Technical advances in recent years have enabled comprehensive genetic analyses that have greatly increased our knowledge of the pathogenesis of human cancers. Various molecular targets have been identified by genetic studies and the subsequent development of compounds targeting altered molecules and pathways has revolutionized cancer therapy.

A caveat of sophisticated genetic assays is that they frequently require high-quality DNA. Most large-scale genetic studies performed to date have thus relied on fresh-frozen material. However, fresh-frozen material is rarely available, the majority of tumor tissue is formalin-fixed and paraffin-embedded (FFPE), which is used for histopathological analysis and allows tissue to be conveniently stored with minimal decay for many years. Vast collections of FFPE tissues have been compiled over the last decades, however, are generally not accessible to modern genetic studies. The low quality and quantity of DNA obtained from FFPE material has impeded large-scale analyses, such as genome sequencing and RNAseq approaches [1, 2]. Decay during fixation and long-term storage of FFPE tissues results in DNA molecules being fragmented and chemically modified, e.g. by hydrolytic damage and crosslinks interconnecting DNA strands as well as DNA and proteins [1]. Methods that would enable high-throughput sequencing analysis of partially degraded tissues with low amounts of input material (i.e. 5 ng or less DNA) are highly sought after [2–5]. Several studies have applied standard library preparation protocols, e.g. by Illumina and SOLiD, to high amounts (> 1 μg) of FFPE input DNA [6, 7] as well as lower quantities ranging down to 50 ng [8, 9]. Only very occasionally these standard protocols have been applied to even lower amounts of FFPE DNA down to 5 ng [5, 10].

The majority of library preparation methods utilize double-stranded DNA. To enable analysis of damaged and fragmented DNA from fossils, these methods have been improved to work with subnanogram amounts of extracted DNA, allowing highly multiplexed target capture and sequencing of ancient DNA. The most recent development is a library preparation specifically targeting single-stranded DNA [11]. Only using this newly developed protocol enabled the genome of an ancient hominin specimen to be sequenced to high coverage, which had not been possible before due to low complexity, i.e. insufficient numbers of unique DNA molecules, in the sequencing libraries [11, 12].

Hence, this single-strand DNA library preparation method may also be beneficial for working with FFPE material as the protocol is optimized for highly fragmented DNA. Furthermore, FFPE-isolated DNA may be expected to be at least partially single-stranded, as isolation protocols generally apply heat to reverse the crosslinking of DNA and proteins.

In this study we test and compare two double-strand library preparation methods, one specifically designed and developed for ancient DNA [13] (hereafter called “MPI”) as well as a commercially available kit from New England Biolabs (hereafter called “NEB”) advertized to require only 5 ng of input DNA, to the single-strand preparation protocol [11]. All three methods were applied to DNA extracts derived from four to 19-year old melanoma FFPE tissue sections. Yield is first quantified in terms of total molecule numbers in the sequencing libraries. A subset of the libraries was then subjected to whole genome shotgun sequencing on an Illumina HiSeq or MiSeq instrument to further characterize the library molecules with respect to sequence complexity, molecule length distribution, base composition and potential damage and fragmentation patterns. Lastly, whole exome enrichment and sequencing was performed with FFPE DNA extracted from an array of different cancer types. Collectively these data will allow a comprehensive evaluation of the methods with respect to their suitability for routine processing of cancer FFPE DNA in large-scale studies, e.g. aiming at genome wide mutational profiling of various cancers from scarce input material like biopsies and histological sections.

## RESULTS

### Yield of library preparations

**Figure 1:**
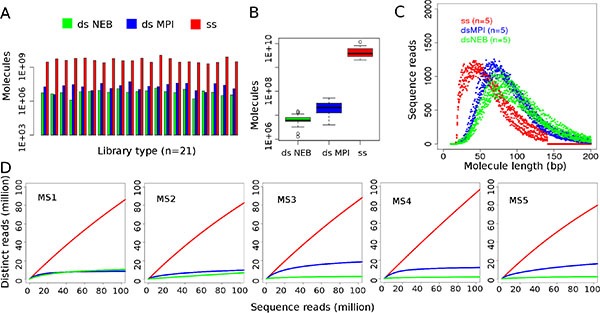
Comparison of double-strand and single-strand libraries (**A**) Overall copy number yield of library preparation measured in digital droplet PCR. (**B**) Comparisons of copy number yield. Single-strand (ss) library preparation outperforms both double-strand (ds) methods (*P* = 3.716 × 10^−12^ for both, ss vs. dsNEB and ss vs. dsMPI comparisons). Custom ds library preparation (dsMPI) has higher yield than the commercial method (dsNEB, *P* = 7.995 × 10^−06^ for dsNEB vs. dsMPI). Raw *p*-values from two-sided Wilcoxon rank sum test (*n* = 21 samples per library preparation). (**C**) Molecule length of 100,000 unique, mapped reads in single-strand and double-strand libraries. As merged reads were analyzed, the plot displays reads with a maximum length of 141 bp (2 × 76 bp reads before overlap merging) for ss libraries. (**D**) Estimated complexity of single-strand libraries (ss) is higher than of double-strand (ds) libraries.

Three library preparation methods were tested on a set of 21 melanoma FFPE DNA samples and yielded a total of 2.04 × 10^9^–1.16 × 10^10^ molecules for the single-strand method, 3.94 × 10^6^–5.22 × 10^7^ molecules for the MPI double-strand method and 1.26 × 10^6^–1.44 × 10^7^ molecules for the NEB double-strand method (Table 1, Figure 1A). Library preparations with the single-strand method thus yielded between 68 and 5,018 times more library molecules than either of the double-strand methods. These differences were highly significant for both comparisons (two-sided Wilcoxon rank sum tests, *P* = 3.716 × 10^−12^, *n* = 21, Figure 1B). The difference between both double-strand methods was less pronounced, however, the MPI method performed significantly better than the NEB kit (*P* = 7.995 × 10^−06^). When comparing both library types it was apparent that single-strand libraries consist of shorter molecules than double-strand libraries (Figure 1C). Negative controls carried through library preparation and sequencing showed orders of magnitude lower molecule numbers, approximately ten times less reads after sequencing and a lower percentage of mapped unique reads than authentic samples (10% vs. on average 61% in genomic libraries and 1% vs. on average 17% in exome libraries, Tables 2 and 3).

**Table 1:** Samples and library preparation yield

Sample No.	Cancer	FFPE storage (years)	Library molecules ds NEB	Sequencing library	Library molecules ds MPI	Sequencing library	Library molecules ss	Sequencing library	Times more molecules ss vs. ds NEB
1	Melanoma	7	5.58E + 06		1.87E + 07		2.90E + 09	A5347	519
2	Melanoma	8	4.58E + 06		3.94E + 06		5.60E + 09	A5348	1223
3	Melanoma	7	5.70E + 06		2.38E + 07		4.10E + 09	A5349	720
4	Melanoma	9	1.26E + 06		3.04E + 07		6.32E + 09	A5350	5018
NTC1	Water	n.a.	3.40E + 06		8.74E + 05		1.79E + 08	A5351	n.d.
5	Melanoma	8	6.95E + 06		1.88E + 07		6.76E + 09		973
6	Melanoma	7	7.55E + 06		8.92E + 06		4.04E + 09		535
7	Melanoma	7	8.95E + 06		3.78E + 07		2.56E + 09		286
8	Melanoma	8	6.18E + 06		1.36E + 07		6.08E + 09		985
9	Melanoma	9	1.27E + 07		2.46E + 07		3.82E + 09		300
10	Melanoma	8	6.15E + 06		5.22E + 07		3.83E + 09		622
11	Melanoma	4	7.35E + 06		1.12E + 07		6.64E + 09		903
12	Melanoma	6	8.40E + 06		2.10E + 07		2.83E + 09		337
13	Melanoma	8	1.44E + 07		1.96E + 07		1.16E + 10		810
14	Melanoma	9	7.30E + 06		3.16E + 07		5.96E + 09	A8244	816
15	Melanoma	4	5.73E + 06		4.02E + 07		2.85E + 09	A8245	498
16	Melanoma	4	1.14E + 07		3.86E + 07		3.16E + 09	A8246	278
NTC2	Water	n.a.	n.a.		9.60E + 05		4.80E + 07	A8247	n.d.
17	Melanoma	13	1.68E + 06	MS1NEB	8.82E + 06	MS1dsMPI	2.60E + 09	MS1	1550
18	Melanoma	15	7.98E + 06	MS2NEB	6.14E + 06	MS2dsMPI	2.04E + 09	MS2	255
19	Melanoma	17	6.05E + 06	MS3NEB	3.66E + 07	MS3dsMPI	2.72E + 09	MS3	450
20	Melanoma	19	3.23E + 06	MS4NEB	2.56E + 07	MS4dsMPI	8.25E + 09	MS4	2558
21	Melanoma	11	3.65E + 06	MS5NEB	1.27E + 07	MS5dsMPI	3.19E + 09	MS5	873
NTC3	Water	n.a.	5.28E + 05		3.60E + 05		6.65E + 07		
22	Lung cancer	11	n.a.		n.a.		1.44E + 09	A8231	
23	Breast cancer	11	n.a.		n.a.		1.02E + 09	A8232	
24	Colorectal cancer	11	n.a.		n.a.		2.36E + 09	A8233	
25	Prostate cancer	11	n.a.		n.a.		1.25E + 09	A8234	
26	Colorectal cancer	9	n.a.		n.a.		8.65E + 08	A8235	
27	Lung cancer	9	n.a.		n.a.		1.58E + 09	A8236	
28	Breast cancer	9	n.a.		n.a.		6.45E + 08	A8237	
29	Prostate cancer	9	n.a.		n.a.		7.35E + 08	A8238	
30	Lung cancer	6	n.a.		n.a.		2.84E + 09	A8239	
31	Prostate cancer	6	n.a.		n.a.		2.72E + 09	A8240	
32	Colorectal cancer	6	n.a.		n.a.		1.58E + 09	A8241	
33	Breast cancer	6	n.a.		n.a.		2.54E + 09	A8242	
NTC4	Water	n.a.	n.a.		n.a.		6.00E + 07	A8243	

Five ng of FFPE DNA yield higher numbers of molecules in single-strand (ss) than in double-strand (ds) DNA library preparation. Ds MPI: a custom method developed for ancient DNA at the Max Planck Institute EVA Leipzig [13]. Ds NEB: a commercial method from New England Biolabs. N.a. not available. N.d. not determined. NTC. Negative control (also see methods section). For a more detailed overview of the order of sequencing experiments see Supplementary Table S1.

### Sequence complexity

Initial shotgun sequencing of the first four single-strand libraries showed high sequence complexity (100% unique reads for all samples), thus motivating the subsequent comparison to the double-strand library preparation methods (Table 2, Supplementary Table S1, Supplementary Figure S1A). Libraries were then prepared for a second batch of five samples using all three methods. All of those 15 libraries were then shotgun sequenced for comparison. The fraction of unique molecules and thus the complexity estimates of single-strand libraries were higher than those of double-strand libraries (Figure 1D, Table 2). Had the libraries been sequenced further, we estimate coverage would have reached 33–117× for whole genomes and between 17× and > 1,000× for exomes (Tables 2 and 3). The fraction of unique molecules remained stable throughout the sequencing run, allowing an extrapolation beyond the performed sequencing in order to identify rich libraries (Supplementary Figure S1B) [14].

**Table 2:** Sequencing characteristics of genomic single-and double-strand libraries

Sample No.	Library type	Library ID	Mergetrimmed reads	Mapped reads	%	Unique reads	%	Mapped reads of unique	%	Molecules in library	Median molecule length (bp)	Mappable bp	Estimated genome coverage
1	ss	A5347	1539768	1294718	84	1539768	100	1282731	83	2.90E+09	76	1.84E+11	55.64
2	ss	A5348	1255872	1078130	86	1255872	100	1070829	85	5.60E+09	58	2.77E+11	83.92
3	ss	A5349	1318068	1111409	84	1318068	100	1102175	84	4.10E+09	81	2.78E+11	84.15
4	ss	A5350	1607846	1397991	87	1607846	100	1390490	86	6.32E+09	62	3.39E+11	102.69
NTC1	ss	A5351	236065	38093	16	236065	100	23877	10	1.79E+08	n.d.		
17	ss	MS1	4114005	3683777	90	4036964	98	3606736	89	2.60E+09	51	1.16E+11	35.23
18	ss	MS2	3229456	2859033	89	3176913	98	2806490	88	2.04E+09	61	1.08E+11	32.77
19	ss	MS3	3138691	2799387	89	3101849	99	2762545	89	2.72E+09	60	1.44E+11	43.53
20	ss	MS4	3988112	3615532	91	3959556	99	3586976	91	8.25E+09	52	3.86E+11	116.92
21	ss	MS5	3821539	3362766	88	3738118	98	3279345	88	3.19E+09	57	1.56E+11	47.28
17	ds	MS1dsMPI	2868381	1623445	57	2582011	90	1337075	52	8.82E+06	76	3.12E+08	0.09
18	ds	MS2dsMPI	3426906	1902541	56	2723388	79	1199023	44	6.14E+06	86	1.85E+08	0.06
19	ds	MS3dsMPI	3575035	2046427	57	3339449	93	1810841	54	3.66E+07	86	1.59E+09	0.48
20	ds	MS4dsMPI	3400508	2065718	61	3109447	91	1774657	57	2.56E+07	79	1.06E+09	0.32
21	ds	MS5dsMPI	3812279	2181953	57	3304041	87	1673715	51	1.27E+07	82	4.57E+08	0.14
17	ds	MS1NEB	3910758	2194541	56	2785746	71	1069529	38	1.68E+06	88	4.04E+07	0.01
18	ds	MS2NEB	2902274	727263	25	2393922	82	218911	9	7.98E+06	92	5.54E+07	0.02
19	ds	MS3NEB	3870670	2197359	57	2369050	61	695739	29	6.05E+06	99	1.08E+08	0.03
20	ds	MS4NEB	2788557	1631982	59	1622804	58	466229	29	3.23E+06	87	4.70E+07	0.01
21	ds	MS5NEB	3435934	1624254	47	2152694	63	341014	16	3.65E+06	88	3.19E+07	0.01

The percentages of mapped reads are reported without quality filters and before duplicate read removal as well as thereafter for the remaining unique reads. Ss. Single-strand. Ds. Double-strand. NTC. Negative control.

### Patterns of damage in FFPE DNA

**Figure 2:**
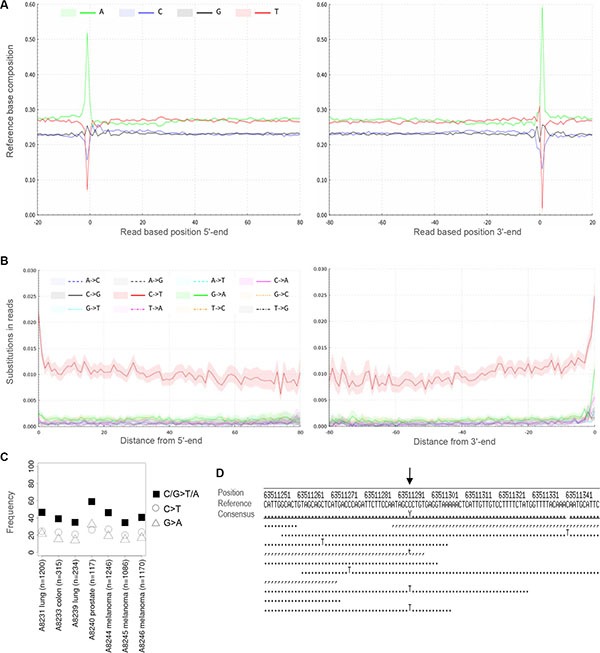
DNA damage in FFPE DNA (**A**) A-fragmentation pattern of FFPE DNA molecules of single-strand library MS1. Frequencies of adenine as the first base of the reference sequence adjacent to the sequenced molecule are observed to be as high as 60%. (**B**) Substitution frequencies throughout the sequenced FFPE DNA molecules. (**C**) C > T and G > A substitution frequencies among called variants. (**D**) Alignment of reads to the human reference genome. C > T damage substitutions in sequencing reads are distinguished from C > T variants (arrow), C > T damage substitutions are dispersed throughout the sequencing reads. C > T variants occur at the same position in a fraction of the sequencing reads. Forward read bases are depicted as points, reverse read bases as commas.

The fixation of tissue with formalin is known to alter the structure of DNA, causing fragmentation and alterations of the organic bases [15–17]. In our sequencing data, reads from FFPE DNA were observed to be short, i.e. mostly below 100 bp (Figure 1C, Supplementary Table S2). When analyzing the mapping positions of our FFPE DNA reads, we observed the first base of the reference genome outside the sequenced molecule to be an adenine in up to 66% of reads with a mean of 53% (Figure 2A, Supplementary Table S2). This pattern reflects a preferential starting point of the sequenced DNA after adenine bases and is hereafter called ‘A-fragmentation’. For double-strand libraries, A-fragmentation is detected as adenines outside the 5′-end and as thymines outside the 3′-end (Supplementary Figure S1C) when analyzing reverse strand molecules.

FFPE DNA showed an elevated frequency of C > T throughout the sequencing reads and most pronounced at the ends of molecules with frequencies up to 3% (Figure 2D, Supplementary Table S2). This pattern was also independent from the tested methods of library preparation and present in single- and double-strand libraries (Supplementary Figure S1D).

### Comparison of damage patterns in single-strand and double-strand libraries

We compared single-strand and double-strand libraries for five FFPE DNA extracts and identified differences in their fragmentation and substitution patterns. The amount of A-fragmentation in single-strand libraries was lower at the 5′-end when compared to corresponding double-strand libraries (*P* = 1.27 × 10^−05^, Supplementary Figure S2). C > T substitutions at both molecule ends reached higher frequencies in single-strand libraries (*P* = 0.02) and the single-strand method utilized shorter molecules (*P* = 2 × 10^−05^).

To test if the FFPE fixation procedure introduces these types of DNA damage, we performed a comparison of FFPE and snap-frozen samples obtained from the same tissues using published raw data [2]. In this study, three prostate tissue samples were taken during surgery and either snap-frozen or formalin-fixed and paraffin-embedded for 3–9 years. All three FFPE samples showed A-fragmentation, unlike their respective snap-frozen counterparts. The latter in fact showed higher frequencies of cytosine residues outside the 5′-ends of molecules and thus, a possible C-fragmentation (Supplementary Figure S3, Supplementary Table S2). Frequencies of C > T substitutions at 5′-ends were slightly increased in FFPE samples in two of three patients, however, not as high as in our long-term stored samples.

It is assumed that longer contact with formalin should increase the amount of DNA damage. Thus we tested whether the incubation time of those tissues in formalin was correlated with the amount of DNA damage observed. For single-strand libraries, we observe increased frequencies of C > T substitutions and shorter molecule length with increasing FFPE storage time (linear models with adjusted R^2^ of 0.3 (5′-end), 0.23 (3′-end), 0.34 (molecule length) and *p*-values of: 0.0035, 0.0112 and 0.0029, respectively, of which the 3′-end loses significance after correction for multiple testing, Supplementary Figure S2).

### Exome sequencing

Five ng of input DNA were retrieved from two 10 μm-sections per FFPE tissue and used to generate single-strand libraries. For exome sequencing on average 34% of reads mapped on target (Table 3). This may seem low for cancer samples in general, but FFPE DNA is very short and will not be captured as efficiently. Fold-enrichment was observed to be 41 to 55-fold and should be comparable across experiments, even when targeted genomic regions differ in size (Supplementary Table S3). Deeper sequencing could then be used to further increase coverage. As we refrained from deeper sequencing, single nucleotide variants (SNV) were called at low stringency (see methods section) for all exomes that had reached 1–5x average unique read coverage. We detected between 30 and 1,300 coding missense SNV per sample and a number of these variants have previously been reported in various types of cancer [18] (Table 4, Supplementary Table S6). Two types of substitutions, C > T and G > A, have been studied most frequently and were previously supposed to include artifacts caused by DNA damage. For all variants called in the exome data, we ascertained the substitution types (Supplementary Table S4), and found C > T and G > A to be most frequent (Figure 2C). With the two types of substitutions representing around 40% of all variant calls, they resemble the upper range of previously sequenced FFPE and fresh-frozen cancer DNA ranging around 20–40% [8, 19].

**Table 3:** Sequencing characteristics of single-strand exome libraries

Sample No.	Library ID	Mergetrimmed reads	Mapped reads on genome	%	Mapped reads on target	%	Unique reads	%	Mapped reads on target of unique	%	Molecules in library	Median molecule length (bp)	Mappable bp	Estimated exome coverage
22	A8231	10400578	8880352	85	3664128	35	9027766	87	2694184	30	1.44E + 09	72	2.69E + 10	814
23	A8232	13928657	8590615	62	4673607	34	7675101	55	4673607	61	1.02E + 09	57	1.95E + 10	591
24	A8233	14725566	11548822	78	5440538	37	9148314	62	1400802	15	2.36E + 09	64	1.43E + 10	434
25	A8234	13510755	9111893	67	4503396	33	7794873	58	445740	6	1.25E + 09	60	2.47E + 09	75
26	A8235	13878538	9302472	67	4884491	35	7652137	55	434072	6	8.65E + 08	59	1.60E + 09	48
27	A8236	12960033	9510227	73	4625240	36	7428921	57	652190	9	1.58E + 09	62	4.91E + 09	149
28	A8237	14125480	8905778	63	4674789	33	8020581	57	344949	4	6.45E + 08	59	9.29E + 08	28
29	A8238	13978045	8283185	59	4598067	33	7644855	55	190019	2	7.35E + 08	57	5.70E + 08	17
30	A8239	12035341	8406977	70	3801180	32	8220549	68	1002280	12	2.84E + 09	71	1.68E + 10	509
31	A8240	10933844	6549725	60	3043865	28	7709969	71	698527	9	2.72E + 09	71	1.23E + 10	374
32	A8241	11869932	7089421	60	3557527	30	7483786	63	408108	5	1.58E + 09	65	3.52E + 09	107
33	A8242	11869182	7085969	60	3872693	33	6586746	55	203156	3	2.54E + 09	55	2.39E + 09	72
NTC4	A8243	1588390	394319	25	114244	7	1327044	84	10681	1	6.00E + 07	n.d.	n.d.	n.d.
14	A8244	10484892	9184487	88	4079825	39	8752541	83	2839016	32	5.96E + 09	75	1.21E + 11	3668
15	A8245	9956899	8806918	88	3742592	38	8234199	83	2480366	30	2.85E + 09	86	6.11E + 10	1850
16	A8246	13621017	11778127	86	5246793	39	10605136	78	3062774	29	3.16E + 09	71	5.04E + 10	1529
NTC2	A8247	1308857	295176	23	117051	9	1098888	84	8913	1	4.80E + 07	n.d.	n.d.	n.d.

N.d. not determined. NTC. Negative control.

**Table 4:** Coding missense SNV called in exome sequencing data

Library and cancer	Chr	Start	Ref	Alt	Coverage	Variant reads	Freq	Gene	Amino acid change	COSMIC IDs; occurence
A8231	1	115256529	T	C	10	6	0.6	NRAS	NRAS:NM_002524:exon3:c.A182G:p.Q61R	584;49(NS),12(lung), and more
Lung	2	179469536	C	A	11	7	0.64	TTN	TTN:NM_003319:exon109:c.G27085T:p.G9029C	718146,718147,718144,1148633,718145;1(lung)
	7	23390935	C	A	14	4	0.29	IGF2BP3	IGF2BP3:NM_006547:exon6:c.G672T:p.Q224H	287378;1(large_intestine)
	17	39296361	A	G	24	11	0.46	KRTAP4-6	KRTAP4-6:NM_030976:exon 1:c.T379C:p.S127P	1479552;2(breasl),1(lung)
	18	63511294	C	T	8	3	0.38	CDH7	CDH7:NM_004361: exon7:c.C1228T:p.P410S	1523528,1523527;1(lung)
A8233	1	145248876	A	G	18	4	0.22	NOTCH2NL	NOTCH2NL:NM_203458:exon2:c.A20G:p.N7S	291720,291719;1(large_intestine)
Coloon	5	833915	G	T	8	4	0.5	ZDHHC11	ZDHHC11:NM_024786:exon7:c.C908A:p.A303D	131334,131335;1(liver),4(prostate)
	3	195452799	C	T	8	4	0.5	MUC20	MUC20:NM_001282506:exon2:c.C1325T:p.T442I	149548;1(stomach)
	14	20296004	C	T	9	5	0.56	OR4N2	OR4N2:NM_001004723:exon2:c.C397T:p.P133S	147728;1(stomach)
	14	19571357	T	C	10	2	0.2	POTEG	POTEG:NM_001005356:exon7:c.T1136C:p.L379S	1477410;3(breast)
A8239	1	146458025	T	C	33	10	0.3	NBPF10	NBPF10:NM_001039703:exon78:c.T9751C:p.Y3251H	1320027;1(ovary)
Lung	7	151927021	C	A	10	3	0.3	KMT2C	KMT2G:NM_170606:exon18:c.G2963T:p.C988F	150426,150427;1(stomach)
	9	69423641	T	G	8	4	0.5	ANKRD20A4	ANKRD20A4:NM_001098805:exon15:c.T1937G:p.M646R	1490069;1(breast)
A8240	14	19571357	T	C	8	2	0.25	POTEG	POTEG:NM_001005356:exon7:c.T1136C:p.L379S	1477410;3(breast)
Prostate	2	107049714	C	G	9	4	0.44	RGPD3	RGPD3:NM_001144013:exon16:c.G2233C:p.E745Q	1526982,1526983;1(lung)
	7	151962265	C	T	10	2	0.2	KMT2C	KMT2C:NM_170606:exon8:c.G1042A:p.D348N	228111,228110;1(skin)
A8244	1	145248876	A	G	18	2	0.11	NOTCH2NL	NOTCH2NL:NM_203458:exon2:c.A20G:p.N7S	291720,291719;1(large_intestine)
Melanoma	1	145281656	A	T	19	2	0.11	NOTCH2NL	NOTCH2NL:NM_203458:exon4:c.A586T:p.T196S	1179143,1179144;1(prostate),1(central_nervous_system)
	13	103701690	G	A	9	5	0.56	SLC10A2	SLC10A2:NM_000452:exon5:c.C868T:p.P290S	1666308;1(eye)
	20	1902301	G	A	13	2	0.15	SIRPA	SIRPA:NM_001040023:exon3:c.G697A:p.V233I	1647366,723497;1(prostate),3(lung)
	X	140993885	C	T	8	4	0.5	MAGEC1	MAGEC1:NM_005462:exon4:c.C695T:p.P232L	226892;1(skin)
A8245	19	7810517	T	A	15	3	0.2	CD209	CD209:NM_001144894:exon2:c.A503T:p.Q168L	221848,221847;1(oesophagus),1(skin),1(cervix)
Melanoma	19	7810586	T	A	17	2	0.12	CD209	CD209:NM_001144894:exon2:c.A434T:p.Q145L	221850,221849;1(skin)
	7	76126737	C	T	9	4	0.44	DTX2	DTX2:NM_001102595:exon5:c.C1093T:p.R365C	300761;1(ovary),1(large_intestine), and more
	9	21239504	T	C	24	6	0.25	IFNA14	IFNA14:NM_002172:exon1:c.A431G:p.K144R	403874;1(lung)
A8246	1	152187935	C	T	30	5	0.17	HRNR	HRNR:NM_001009931:exon3:c.G6170A:p.R2057Q	224173;1(skin)
Melanoma	12	11461549	G	C	19	2	0.11	PRB4	PRB4:NM_002723:exon3:c.C368G:p.P123R	1628396;1(liver)
	12	31250875	G	C	9	4	0.44	DDX11	DDX11:NM_001257144:exon18:c.G1819C:p.A607P	304699,1318021;1(lung),1(large_intestine), and more
	12	52699033	G	A	10	3	0.3	KRT86	KRT86:NM_002284:exon5:c.G745A:p.V249I	404426;1(lung)
	15	78290635	C	T	8	3	0.38	TBC1D2B	TBC1D2B:NM_015079:exon13:c.G2707A:p.D903N	458956,86571;2(NS),2(urinary_tract), and more
	5	23526987	C	G	23	6	0.26	PRDM9	PRDM9:NM_020227:exon11:c.C1790G:p.T597S	231055;1(skin),2(prostate),1(large_intestine)

Subset of variants detected in this study and also detected previously in cancer sequencing [18]. Full lists of called variants are listed in Supplementary Table S6. Freq: frequency.

The GC content of our genomic single-strand libraries ranged from 41–47%, which was slightly lower than that of double-strand libraries ranging from 46–50% and thus closer to the known GC content of the human genome around 42% (Supplementary Table S5). Single-strand exome libraries had a higher GC content of 45–51%, likely reflecting a preference of higher GC molecules in hybridization capture.

## DISCUSSION

In this study, we successfully tested a single-strand DNA library preparation method, originally established for ancient DNA, to generate genome wide sequencing data of FFPE derived cancer DNA. A comparison with standard double-strand methods showed that single-strand library preparation is far superior to previously reported methods of analyzing FFPE-isolated DNA. Complexity of sequencing libraries is a critical issue when applying high-throughput sequencing to scarce FFPE samples [5]. Genome sequencing requires high complexity, meaning sufficient numbers of distinct, or unique, molecules, especially when increasing sequencing depth is desired. With 98–100% unique molecules in our genomic libraries the single-strand method outperforms two double-strand methods (58–93%), one developed for ancient DNA and the other specifically advertised to cope with low-quantity samples. Complexity of single-strand libraries was also considerably higher than that reported for alternative double-strand protocols by Illumina and SOLiD with 35–75% [3] and 60–63% [19] unique molecules, respectively. Especially after hybridization-based whole exome capture, the sequence complexity further decreases owing to the additional handling steps. Therefore, the complexity achieved in the initial library preparation is of critical importance. Consequently, our comparisons show that the single-strand libraries contain higher complexity after the exome enrichment, namely 55–87% unique molecules, compared to previously reported double-strand library preparations of Illumina protocols with 15–62% [20], 40–62% [2], 7–88% [5] and SOLiD protocols with ∼50% [6] of unique molecules. Notably, other studies reportedly required hundreds of nano- or even micrograms of input DNA [2, 3, 6, 20]. Some previous studies using lower input amounts of FFPE DNA do not report sequence complexity. Between five and 50ng of input DNA were used to e.g. generate copy number karyograms with ∼60% of reads mapped, however, the percentage of unique reads was not reported [4]. While input amounts of ≤ 250 ng FFPE DNA were previously reported as insufficient for adequate exome coverage [10], recently, ‘successfully’ sequenced exomes have been reported from as little as 16 ng input FFPE DNA in a large study of 99 FFPE samples [5]. In the same study, duplicate molecules in double-strand libraries were found to be problematic, requiring high amounts of additional sequencing to reach a sufficient unique sequencing coverage [5]. Eventually, such libraries had been discarded. Our study demonstrates that those hurdles can be overcome by employing the single-strand library preparation protocol to retrieve molecule counts sufficient for whole genome and exome sequencing at adequate unique molecule depth. We measure factors that allow us to estimate the anticipated coverage in order to conclude which samples are worthwhile sequencing. The pronounced gain of molecules in single-strand libraries appears to be at least partially the result of utilizing shorter molecules [11]. We observed a difference of over 20 bp in molecule length between single-strand (61.5 bp) and double-strand (86.5 bp) libraries (Supplementary Figure S2E). Hence, some of the gain of molecules is lost after sequencing, as very short molecules cannot be mapped accurately to the human reference genome and are thus excluded from the subsequent analysis. However, the percentage of mapped reads in genomic single strand libraries was on average 87% and with high complexity and molecule numbers these libraries are estimated to yield between 33x and 117x unique read whole genome coverage (Table 2). The targeted exome single-strand libraries had on average 71% mapped reads and are estimated to yield 17x–3,668x fold exome coverage. Thus, in single-strand libraries a loss of molecule length is well compensated by molecule numbers and complexity. On the contrary for double-strand libraries, which had lower molecule numbers and complexity, we also observed lower percentages of mapped reads, on average 48%. This factor may be an underestimate, as it should usually range higher. However, even with all molecules mapped, the double strand libraries would reach only 0.3x–0.89x genome coverage, which is insufficient for most applications.

The proportion of single-stranded DNA in DNA extracts likely benefits from heating steps during DNA extraction. The DNeasy Blood&Tissue kit (Qiagen) is a widely used standard with moderate heating at 56°C. Other extraction kits vary in heating temperature and duration. Most kits use a proteinase K digestion at ≥ 37°C, others are using higher temperatures up to 90°C combined with the addition of heated elution buffer to the spin column (Qiagen AllPrep Micro and DNA/RNA FFPE kit). In our case, the DNA Mini kit (Qiagen) was used and contains a 56°C and a 70°C incubation. The benefit shown for the single-strand over the double-strand library preparation may be even further increased when incubations are carried out at higher temperatures. However, DNA extraction procedures should be further optimized for the retrieval of single-strand DNA.

Fragmentation of FFPE DNA has previously been analyzed with capillary gel electrophoresis and average molecule length was found to range between 20 and 100 bp in DNA extracts isolated from FFPE tissues stored between 8 and 48 years [15]. Determining the length of molecules via sequencing we observe similar molecule lengths in our samples that have been stored 4–19 years (Figure 1, Supplementary Figure S4A and 4B). We analyzed fragmentation in the sequence context and found that most often the sequenced molecules start and end next to an adenine residue in the reference genome. As this A-fragmentation pattern was observed in our data obtained from single-strand and double-strand libraries, as well as in published data from double-strand libraries, we are confident it represents a ubiquitous pattern in FFPE DNA. A-fragmentation probably results from the process of interstrand crosslinking during formalin fixation. Formalin has been reported to introduce interstrand crosslinks preferentially at adenine residues [17, 21]. During DNA extraction, DNA strands presumably break adjacent to these crosslinks and thus preferentially release DNA molecules between adenines.

Relatively frequent events in formalin treated DNA are intrastrand crosslinks at GG-CC and GA-TC sites which, if repaired lead to tandem base substitutions [22]. These types of mutation supposedly make up about half of the formalin-induced mutations. However, DNA strands with intrastrand crosslinks presumably will not be available for library preparation, and the absence of DNA repair in fixed tissues may explain why these patterns are not obvious in our data. The same applies to G > T transversions, previously reported to be one of the most frequent single base substitutions [22]. Only DNA repair will change the N-2-hydroxymethyl adduct to guanine into a G > T transversion, which we consequently do not see elevated in formalin-fixed tissues. It is in general not clear if some of the true damage patterns of the molecule ends may be altered during sequence library preparation. Furthermore, unknown biases may exist in ligation efficiency at the molecule ends depending on the particular base composition.

The diversity of DNA alterations introduced by FFPE storage is not yet fully understood. We observed a potential C-fragmentation in the snap-frozen sample dataset (Supplementary Figure S3). Some FFPE libraries, A8239 and A8240, a lung and prostate cancer, further showed high guanine frequencies at the first base outside the sequenced molecule (Supplementary Figures S4C and S4D) and thus a potential G-fragmentation or depurination as described for ancient DNA [23, 24]. These results indicate a number of yet unknown patterns of DNA damage introduced during freezing, fixation, extraction and library preparation.

The deamination of C > T is a well-studied process that is known to increase the substitution frequencies of C > T in DNA sequences [23, 25–28]. With a frequency of around 1% within sequenced molecules and up to 3% at their ends, FFPE DNA contains lower frequencies of C > T damage than generally observed in ancient DNA, where frequencies often exceed 20–40% at molecule ends, e.g. in the genomes of Neanderthals and the Tyrolean Iceman [23, 24]. Our data reveal elevated frequencies of C > T, however, not G > A substitutions in FFPE DNA, which have previously been reported in sequences of PCR products and double-strand libraries [8, 9, 19, 20, 29]. PCR and double-strand library preparation often do not represent the original 3′-end of forward strand molecules due to a blunt ending reaction or by copying the opposite 5′-strand during the process of library preparation. G > A damage substitutions in sequencing reads are thus thought to represent reverse complements of the underlying cytosine deamination in the 5′-end of reverse strands of the original molecule [20, 23].

Comparing published DNA sequencing data from FFPE and frozen samples indicated that A-fragmentation and C > T substitutions are introduced by the FFPE fixation procedure. An analysis of samples with increasing storage time indicates that damage accumulates with FFPE storage time, as suggested previously [20, 30]. Possible batch effects of the fixation procedures performed many years ago cannot be excluded. To understand the relationship of FFPE fixation and DNA degradation in more detail, experiments with a prospective design including tests with varying formalin concentrations and fixation times, would be necessary.

Our data revealed diverse DNA alterations introduced by FFPE storage conditions of patient material. Presumably, previous studies describing mutational signatures across human cancer types have also contained a fraction of samples derived from FFPE material [31, 32], summarized at http://cancer.sanger.ac.uk/cosmic/signatures. Thus, understanding damage patterns in FFPE-derived DNA is of critical importance to distinguish and decipher the mutational signatures described for any given type of cancer.

In the past, elevated frequencies of non-reproducible substitutions have been reported for FFPE DNA and were long argued to be a major drawback of this material. However, frequent C > T and G > A transitions were originally detected in experiments based on PCR and Sanger sequencing, where single or few damaged molecules have been amplified [29]. Using current high-throughput sequencing techniques, the impact of single damaged molecules for variant calling and hence for the detection of mutations is much less problematic. Moreover, recent studies repeatedly showed a high concordance of variants called and similar mutational profiles both in frozen and FFPE tissues [5, 8, 9, 20], which can be explained by the low frequency of C > T damage in sequenced molecules. We estimate that as a result of DNA damage, at any sequenced position about 1% of sequencing reads show a C > T substitution (Figure 2B), which is comparable to previously reported data [19]. In standard genome or exome sequencing with e.g. 30–80x coverage, 1% of sequencing reads would correspond to single reads carrying C > T damage substitutions. However, reads of both sequencing directions should be required routinely to support variant calls as exemplified in Figure 2D. Moreover, variants are routinely reported to clinicians only when observed in a pre-determined minimal fraction, e.g. 5% of sequencing reads. The observed frequencies of FFPE damage C > T substitutions will therefore remain below this threshold in the majority of cases and would thus only rarely cause false positive variant calls. Assuming an underlying Poisson distribution, we estimate up to around 2% of variant calls may represent false positive findings (Supplementary Figure S5), which is concordant with previous results on FFPE exome sequencing of paired fresh-frozen and FFPE samples. False positive mutation calls were identified from FFPE GIST tumor samples using frozen and peripheral blood samples as controls [9]. False positives were observed in the range of 0–1% of all mutation calls for three high-quality FFPE samples and at 2% for one low-quality FFPE sample. Taking the step from sequencing reads to variant calls, previous studies notably showed similar substitution frequencies within variants called from FFPE and fresh-frozen cancer DNA. For the more frequent substitution types C > T and G > A previous estimates ranged around 20–40% [8, 19]. At low coverage we observe similar frequencies of C > T and G > A substitutions, around 40% and higher, in the variants called from single-stranded FFPE DNA of lung, colon, prostate and skin cancers (Figure 2C). As there is no obvious GC bias in single-strand libraries (Supplementary Table S5 and Supplementary Figure S6), we attribute these higher frequencies of C > T and G > A to the relatively shallow sequencing performed here. In future, detailed studies comparing the single-strand method on paired samples of frozen and FFPE material are needed. However, the identified variants in our exome sequencing of single-strand libraries revealed characteristic mutations that have previously been described for cancer DNA (Table 4, Supplementary Table S6). In summary, these data indicate that the slightly elevated frequency of C > T in FFPE DNA will not be detrimental to routine high-throughput sequencing in the clinical setting.

Most large-scale genetic research efforts in oncology to date have been required to rely on fresh-frozen material, as results obtained from FFPE tissue were poor. Many genetic studies have been impeded owing to insufficient or inadequate tumor tissue availability. As FFPE material has been the standard for archiving tumor tissues for decades, access to fresh-frozen material remains an exception in a routine clinical setting. Allowing large-scale genetic analyses based on the vast collections of FFPE material, we expect the single-strand library preparation method to greatly facilitate future research, especially in the field of molecular oncology.

In the era of personalized medicine, analyzing a patient's individual tumor can be critical to determine genetic alterations relevant in terms of prognosis and therapy decisions. Especially in a clinical oncology setting, the effective use of DNA retrieved from FFPE or biopsy material is crucial, in particular for patients where additional tumor tissue is generally not available or obtaining it would require additional surgery.

In summary, our analyses indicate that the number of unique molecules obtained in double-strand sequencing libraries has hampered large-scale sequencing studies of FFPE DNA. Higher yield and sequence complexity can be obtained from the same source material using a single-strand library preparation protocol, which makes whole exome and genome sequencing feasible from only 5 ng of FFPE-isolated DNA, generally corresponding to one or two 10 μm-sections of FFPE tissue. Although the generated sequence data contain C > T DNA damage as well as strand breaks adjacent to adenines, we do not expect variant calling to be compromised if quality criteria, such as minimum bidirectional read coverage, are applied. Given the significant improvements over conventional double-strand protocols for high-throughput sequencing of FFPE DNA, we believe the single-strand library preparation protocol will be of great value to clinicians and researchers alike.

## MATERIALS AND METHODS

### Samples

Melanoma samples were obtained from the Skin Cancer Biobank (SCABIO), Department of Dermatology, University Hospital Essen. Samples of patients with lung, breast, colorectal and prostate cancer were obtained and anonymized at the Department of Pathology, University Hospital Carl Gustav Carus, Technical University Dresden. Tumor samples were collected during the surgery, subsequently formalin-fixed and paraffin-embedded and stored as FFPE blocks at room temperature. To study the effect of sample storage time we obtained data on the year of surgery and fixation of the sample as well as date of DNA extraction from SCABIO, University Hospital Essen and the regional clinical cancer registry Dresden.

### Ethics statement

Participants consented to their biological material being used for diagnostic purposes, and remainders of the material were used in this study. The study was approved by the respective ethics committees of the faculties for medicine of the University Duisburg-Essen (study ID 11-4715) and the Technical University Dresden (study ID EK59032007), Germany. The dataset presented here is anonymized in a manner that beyond the year of surgery and the type of cancer no personal information is given for study participants.

### DNA extraction

DNA extraction from tumor samples and corresponding blood was performed with the QIAamp DNA Mini kit (Qiagen, Hilden, Germany). This standard procedure includes incubation in xylene for deparaffinization and incubation with proteinase K at 56°C as well as an incubation step at 70°C for ten minutes.

### Library preparation, quantification, amplification and enrichment

DNA libraries were prepared from a total of 5 ng of extracted genomic DNA. Prior to the library preparation a stock of 5 ng/ul was prepared and aliquoted to ensure equal input quantities in all preparations. Libraries were prepared using two previously published custom protocols especially optimized for ancient and degraded DNA, i.e. one for double-stranded DNA [13] and one for single-stranded DNA [11], as well as a commercially available kit - NEBNext^®^ Ultra™ DNA Library Prep Kit (New England BioLabs, Ipswich, MA) advertised to be capable of using DNA input amounts as low as 5 ng total. The double-strand DNA method was implemented as described in the original publication. The NEB kit was used according to the manufacturers recommendations, but with a 1:30 dilution of the adapters in the ligation step to reduce the otherwise extensive formation of adapter dimers. For the single-strand method we followed the protocol as published [11] including the preparation and aliquoting of all chemicals in a dedicated pre-PCR facility, and with a modification reported recently by Korlevic *et al.* [33]: the single-stranded adaptor oligonucleotide (CL78) was cleaned from human contamination and synthesis artifacts by *E. coli* exonuclease I treatment. For this purpose, 7.5 μM of oligonucleotide was incubated for 20 min at 37°C in 1×CircLigase II buffer with 46 U exonuclease I (New England BioLabs, Ipswich, MA) in a reaction volume of 23 μl. The exonuclease was then heat-inactivated for 1 min at 95°C. One microliter of each library was used to measure the number of library molecules by digital droplet (dd)PCR (QX200 system; Bio-Rad) using primers IS7 and IS8 [13] and EvaGreen chemistry following the manufacturer's recommendations. When necessary, dilutions were measured. The total number of library molecules was then calculated for the full volume of each library (20 ul for the two MPI methods and 50 ul for the NEBNext kit). The remaining libraries were amplified and tagged with pairs of sample-specific barcodes [34] using AccuPrime Pfx DNA polymerase (Life Technologies, Carlsbad, CA) as described elsewhere [35], but using higher primer concentrations (1 μM).

### Negative controls

Water was used in negative controls and carried through all steps of the library preparation until the ddPCR and partly also to sequencing. Molecule numbers in negative controls were orders of magnitude lower than samples, with the exception of one NEB double-strand preparation (NTC1 in Table 1). It has been reported that standard library preparation methods (e.g., Illumina's TruSeq DNA sample preparation kit, no. 15026486 Rev. A, or NEB's NEBNext Ultra DNA library preparation kit, v1.1) are less suitable for the generation of libraries from highly degraded and low-quantity DNA because a background of around 1 × 10E^8^ molecules may be created mostly derived from adapter dimers and synthesis artifacts present in the adapter oligonucleotide [11]. The number of sequencing reads and mapped reads was assessed for three negative controls of single-strand library preparation.

### Exome capture

Two 10 μm-sections of ∼25 square mm per FFPE tissue were used for DNA extraction and 5 ng of the extracted DNA was used to prepare single-strand DNA libraries. For exome capture, 4 ul of libraries prepared from single-stranded DNA were used for hybridization capture as described previously [36]. To more efficiently enrich for the coding fraction of the genome, two consecutive rounds of hybridization captures were carried out.

### Sequencing and sequence analyses

Sequencing was performed on Illumina MiSeq and HiSeq instruments with parameters outlined in detail in Supplementary Table S1. Base calling was performed by Bustard (Illumina Corp.) for MiSeq data and by freeIbis [37] for HiSeq data. Illumina adapters were removed, putative chimeric sequences were flagged as failing quality control and overlapping paired reads were merged using leeHom with the “—ancientdna” option [38]. Reads were assigned to their sample of origin using deML [39] and jivebunny with default quality thresholds (https://github.com/udo-stenzel/biohazard). Reads were then mapped to the human genome reference hg19 using bwa-0.4.9. Library complexity was estimated from the sequencing reads using preseq-0.1.0 [14] with steps of 100,000 reads. Duplicate reads were marked using Picardtools (http://picard.sourceforge.net/) and the mapped fraction of reads was calculated without read mapping quality filters requiring at least one of the read segments mapped. The GC content was determined for unique mapped reads. Distributions of molecule length in sequencing libraries of ≥ 10 bp were determined using the “–m” option implemented in samtools (https://github.com/mpieva/samtools-patched) from 100,000 merged, unique and mapped reads and the median was calculated.

Estimates for genome and exome coverage were calculated as follows. Mappable bp were calculated by the fraction of unique reads multiplied with the fraction of mapped (unique) reads and multiplied with the total molecule count and with the median molecule length. We then extrapolated to mappable genomes and exomes. Substitution frequencies and the reference base composition were analyzed from 100,000 mapped reads of a minimum length of 30 bp using damage patterns (https://github.com/udo-stenzel/damage-patterns). We additionally analyzed publicly available sequencing data for FFPE and corresponding snap-frozen tissues obtained via ICGC (accession EGAD00001000033, DACO-1002654). For these single end reads, information was available only for the 5′-end.

For exome sequencing data the average coverage over the entire bed file of target regions was determined using bedtools [40]. The numbers of unique reads mapping to the entire genome and to the target region were determined using samtools (https://github.com/mpieva/samtools-patched) and requiring a mapping quality of 20.

SNV calling was performed using samtools-1.0 [41]. Variant calls required one unique read for each variant allele in forward and reverse direction, respectively, a coverage of at least 8 unique reads and a variant frequency of at least 10%. Annovar [42] was applied to identify nonsynonymous coding SNV not known from dbSNP138 or the 1000 genomes project (MAF > 0.01). SNV located in repetitive regions were excluded using the UCSC repeatmasker track and intersectBed [40]. From all called SNV we identified variants previously detected in cancer sequencing projects by cross referencing the COSMIC database [18]. As we used archived FFPE samples, it was not possible to obtain consent for the publication of the sequencing raw data retrospectively from some study participants as they are deceased. Thus, the sequencing raw data are not made publicly available.

### Statistical analyses

To test whether library preparation yields and amount of DNA damage in different groups of samples varied significantly, we applied two-sided Wilcoxon rank sum tests in R v. 3.0. [43]. Correlations of C > T substitution and A-fragmentation frequencies, as well as molecule length with sample storage time were performed using linear models in R. The probability to observe > 5% of reads with C > T damage was modeled using the Poisson distribution for read coverage up to 100x with an expected frequency of C > T in 1% of reads at any sequenced nucleotide position. The model also required to observe at least two reads with C > T as routinely a minimum of two reads is required to support variant calls.

## SUPPLEMENTARY MATERIALS FIGURES AND TABLES





## References

[1] van Beers EH, Joosse SA, Ligtenberg MJ, Fles R, Hogervorst FBL, Verhoef S, Nederlof PM (2005). A multiplex PCR predictor for aCGH success of FFPE samples. Br J Cancer.

[2] Kerick M, Isau M, Timmermann B, Sultmann H, Herwig R, Krobitsch S, Schaefer G, Verdorfer I, Bartsch G, Klocker H, Lehrach H, Schweiger M (2011). Targeted high throughput sequencing in clinical cancer Settings: formaldehyde fixed-paraffin embedded (FFPE) tumor tissues, input amount and tumor heterogeneity. BMC Med Genet.

[3] Schweiger MR, Kerick M, Timmermann B, Albrecht MW, Borodina T, Parkhomchuk D, Zatloukal K, Lehrach H (2009). Genome-Wide Massively Parallel Sequencing of Formaldehyde Fixed-Paraffin Embedded (FFPE) Tumor Tissues for Copy-Number- and Mutation-Analysis. PLoS ONE.

[4] Wood HM, Belvedere O, Conway C, Daly C, Chalkley R, Bickerdike M, McKinley C, Egan P, Ross L, Hayward B, Morgan J, Davidson L, MacLennan K (2010). Using next-generation sequencing for high resolution multiplex analysis of copy number variation from nanogram quantities of DNA from formalin-fixed paraffin-embedded specimens. Nucleic Acids Res.

[5] Allen EMV, Wagle N, Stojanov P, Perrin DL, Cibulskis K, Marlow S, Jane-Valbuena J, Friedrich DC, Kryukov G, Carter SL, McKenna A, Sivachenko A, Rosenberg M (2014). Whole-exome sequencing and clinical interpretation of FFPE tumor samples to guide precision cancer medicine. Nat Med.

[6] Menon R, Deng M, Boehm D, Braun M, Fend F, Boehm D, Biskup S, Perner S (2012). Exome Enrichment and SOLiD Sequencing of Formalin Fixed Paraffin Embedded (FFPE) Prostate Cancer Tissue. Int J Mol Sci.

[7] Wagle N, Berger MF, Davis MJ, Blumenstiel B, DeFelice M, Pochanard P, Ducar M, Van Hummelen P, MacConaill LE, Hahn WC (2012). High-throughput detection of actionable genomic alterations in clinical tumor samples by targeted, massively parallel sequencing. Cancer Discov.

[8] Munchel S, Hoang Y, Zhao Y, Cottrell J, Klotzle B, Godwin AK, Koestler D, Beyerlein P, Fan J-B, Bibikova M, Chien J (2015). Targeted or whole genome sequencing of formalin fixed tissue samples: potential applications in cancer genomics. Oncotarget.

[9] Astolfi A, Urbini M, Indio V, Nannini M, Genovese CG, Santini D, Saponara M, Mandrioli A, Ercolani G, Brandi G, Biasco G, Pantaleo MA (2015). Whole exome sequencing (WES) on formalin-fixed, paraffin-embedded (FFPE) tumor tissue in gastrointestinal stromal tumors (GIST). BMC Genomics.

[10] Beltran H, Yelensky R, Frampton GM, Park K, Downing SR, MacDonald TY, Jarosz M, Lipson D, Tagawa ST, Nanus DM (2012). Targeted Next-generation Sequencing of Advanced Prostate Cancer Identifies Potential Therapeutic Targets and Disease Heterogeneity. Eur Urol.

[11] Gansauge M-T, Meyer M (2013). Single-stranded DNA library preparation for the sequencing of ancient or damaged DNA. Nat Protoc.

[12] Meyer M, Kircher M, Gansauge M-T, Li H, Racimo F, Mallick S, Schraiber JG, Jay F, Prüfer K, de Filippo C, Sudmant PH, Alkan C, Fu Q (2012). A High-Coverage Genome Sequence from an Archaic Denisovan Individual. Science.

[13] Meyer M, Kircher M (2010). Illumina Sequencing Library Preparation for Highly Multiplexed Target Capture and Sequencing. Cold Spring Harb Protoc.

[14] Daley T, Smith AD (2013). Predicting the molecular complexity of sequencing libraries. Nat Methods.

[15] Zimmermann J, Hajibabaei M, Blackburn DC, Hanken J, Cantin E, Posfai J, Evans TC (2008). DNA damage in preserved specimens and tissue samples: a molecular assessment. Front Zool.

[16] McGhee JD, Von Hippel PH (1975). Formaldehyde as a probe of DNA structure. I. Reaction with exocyclic amino groups of DNA bases. Biochemistry.

[17] Huang H, Solomon MS, Hopkins PB (1992). Formaldehyde preferentially interstrand cross-links duplex DNA through deoxyadenosine residues at the sequence 5′-d (AT). J Am Chem Soc.

[18] Forbes SA, Bindal N, Bamford S, Cole C, Kok CY, Beare D, Jia M, Shepherd R, Leung K, Menzies A, Teague JW, Campbell PJ, Stratton MR (2011). COSMIC: mining complete cancer genomes in the Catalogue of Somatic Mutations in Cancer. Nucleic Acids Res.

[19] Yost SE, Smith EN, Schwab RB, Bao L, Jung H, Wang X, Voest E, Pierce JP, Messer K, Parker BA (2012). Identification of high-confidence somatic mutations in whole genome sequence of formalin-fixed breast cancer specimens. Nucleic Acids Res.

[20] Spencer DH, Sehn JK, Abel HJ, Watson MA, Pfeifer JD, Duncavage EJ (2013). Comparison of clinical targeted next-generation sequence data from formalin-fixed and fresh-frozen tissue specimens. J Mol Diagn.

[21] Huang H, Hopkins PB (1993). DNA interstrand cross-linking by formaldehyde: nucleotide sequence preference and covalent structure of the predominant cross-link formed in synthetic oligonucleotides. J Am Chem Soc.

[22] Kawanishi M, Matsuda T, Yagi T (2014). Genotoxicity of formaldehyde: molecular basis of DNA damage and mutation. Front Environ Sci.

[23] Briggs AW, Stenzel U, Johnson PL, Green RE, Kelso J, Prüfer K, Meyer M, Krause J, Ronan MT, Lachmann M (2007). Patterns of damage in genomic DNA sequences from a Neandertal. Proc Natl Acad Sci USA.

[24] Keller A, Graefen A, Ball M, Matzas M, Boisguerin V, Maixner F, Leidinger P, Backes C, Khairat R, Forster M (2012). New insights into the Tyrolean Iceman's origin and phenotype as inferred by whole-genome sequencing. Nat Commun.

[25] Wang TP, Sable HZ, Lampen JO (1950). Enzymatic deamination of cytosine nucleosides. J Biol Chem.

[26] Gilbert MTP, Binladen J, Miller W, Wiuf C, Willerslev E, Poinar H, Carlson JE, Leebens-Mack JH, Schuster SC (2007). Recharacterization of ancient DNA miscoding lesions: insights in the era of sequencing-by-synthesis. Nucleic Acids Res.

[27] Stiller M, Green RE, Ronan M, Simons JF, Du L, He W, Egholm M, Rothberg JM, Keates SG, Ovodov ND (2006). Patterns of nucleotide misincorporations during enzymatic amplification and direct large-scale sequencing of ancient DNA. Proc Natl Acad Sci USA.

[28] Weiss RB, Mineura K, Henderson EE, Duker NJ, DeRiel JK (1983). Enzymic detection of uracil in a cloned and sequenced deoxyribonucleic acid segment. Biochemistry.

[29] Williams C, Pontén F, Moberg C, Söderkvist P, Uhlén M, Pontén J, Sitbon G, Lundeberg J (1999). A high frequency of sequence alterations is due to formalin fixation of archival specimens. Am J Pathol.

[30] Araujo LH, Timmers C, Shilo K, Zhao W, Zhang J, Yu L, Natarajan TG, Miller CJ, Yilmaz AS, Liu T (2015). Impact of Pre-Analytical Variables on Cancer Targeted Gene Sequencing Efficiency. PLoS ONE.

[31] Alexandrov LB, Nik-Zainal S, Wedge DC, Aparicio SAJR, Behjati S, Biankin AV, Bignell GR, Bolli N, Borg A, Børresen-Dale A-L (2013). Signatures of mutational processes in human cancer. Nature.

[32] Nik-Zainal S, Alexandrov LB, Wedge DC, Van Loo P, Greenman CD, Raine K, Jones D, Hinton J, Marshall J, Stebbings LA (2012). Mutational processes molding the genomes of 21 breast cancers. Cell.

[33] Korlevic P, Gerber T, Gansauge M-T, Hajdinjak M, Nagel S, Ayinuer-Petri A, Meyer M (2015). Reducing microbial and human contamination in DNA extractions from ancient bones and teeth. Biotechniques.

[34] Kircher M, Sawyer S, Meyer M (2012). Double indexing overcomes inaccuracies in multiplex sequencing on the Illumina platform. Nucleic Acids Res.

[35] Dabney J, Meyer M (2012). Length and GC-biases during sequencing library amplification: a comparison of various polymerase-buffer systems with ancient and modern DNA sequencing libraries. Biotechniques.

[36] Fu Q, Meyer M, Gao X, Stenzel U, Burbano HA, Kelso J, Pääbo S (2013). DNA analysis of an early modern human from Tianyuan Cave, China. Proc Natl Acad Sci USA.

[37] Renaud G, Kircher M, Stenzel U, Kelso J (2013). freeIbis: an efficient basecaller with calibrated quality scores for Illumina sequencers. Bioinformatics.

[38] Renaud G, Stenzel U, Kelso J (2014). leeHom: adaptor trimming and merging for Illumina sequencing reads. Nucleic Acids Res.

[39] Renaud G, Stenzel U, Maricic T, Wiebe V, Kelso J (2015). deML: robust demultiplexing of Illumina sequences using a likelihood-based approach. Bioinformatics.

[40] Quinlan AR, Hall IM (2010). BEDTools: a flexible suite of utilities for comparing genomic features. Bioinformatics.

[41] Li H, Handsaker B, Wysoker A, Fennell T, Ruan J, Homer N, Marth G, Abecasis G, Durbin R, Subgroup GPDP (2009). The Sequence Alignment/Map format and SAMtools. Bioinformatics.

[42] Wang K, Li M, Hakonarson H (2010). ANNOVAR: functional annotation of genetic variants from high-throughput sequencing data. Nucleic Acids Res.

[43] Team RDC (2013). R: A language and environment for statistical computing. http://www.r-project.org/.

