# Supplementary material for: Single-strand DNA library preparation improves sequencing of formalin-fixed and paraffin-embedded (FFPE) cancer DNA
Source: Oncotarget. 2016 Jul 24;7(37):59115–28. doi: 10.18632/oncotarget.10827 (PMC5312299; doi:10.18632/oncotarget.10827)
Supplement: Supplementary file 1 [file oncotarget-07-59115-s001.pdf]

Single-strand DNA library preparation improves sequencing of formalin-fixed and paraffin-embedded (FFPE) cancer DNA

Supplementary Materials

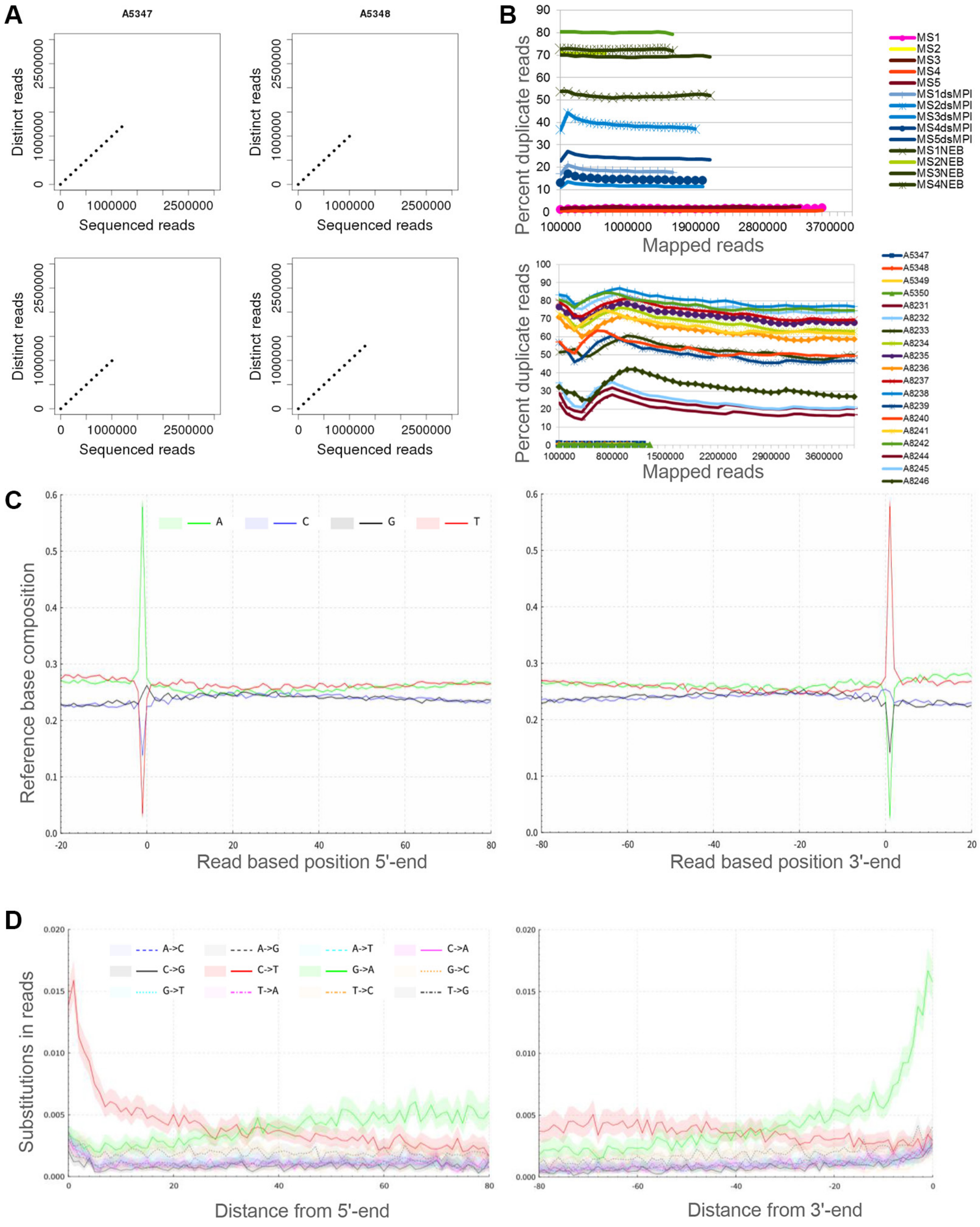

Supplementary Figure S1: Additional information on complexity and damage. (**A**) Observed library complexity of the first FFPE single-strand libraries determined by preseq. (**B**) Percentage of duplicate reads throughout the sequencing given for bins of 100,000 reads. (**C**) A-fragmentation in double-strand library MS1dsMPI. A-fragmentation is detected as adenines outside the 5′-end and as thymines outside the 3′-end. (**D**) Substitutions of C > T are observed at 5′-ends and represented by G > A at 3′-ends.

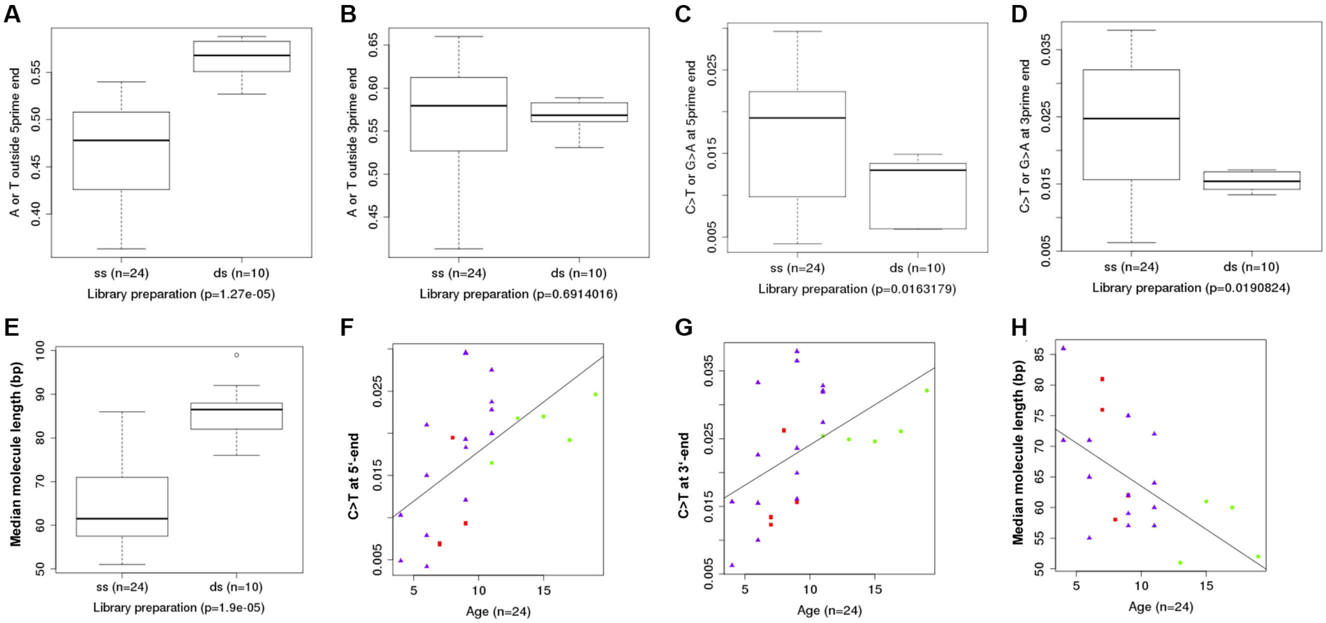

Supplementary Figure S2: Comparison of single-strand (ss) and double-strand (ds) libraries. (**A**) The amount of A-fragmentation at the 5′-end was lower in single-strand libraries when compared to corresponding double-strand libraries (P = 1.27e–05). (**B**) No difference was seen for A-fragmentation at the 3′-end (P = 0.7). (**C** and **D**). C > T substitutions at both molecule ends reached higher frequencies in single-strand libraries (P = 0.02). (**E**) The single-strand method utilized shorter molecules (P = 2e–05). Raw p-values from two-sided Wilcoxon rank tests, comparison A and E remain significant after correcting for five tests when alpha = 0.05. (**F**) Linear models for DNA damage and FFPE storage time (age). Single-strand libraries showed correlation with storage time for increased C > T substitutions at 5′-ends (adj. R^2 = 0.3, p-value = 0.0035), at (**G**) 3′-ends (adj. R^2 = 0.23, p-value = 0.0112) and with (**H**) decreased molecule length (adj. R^2 = 0.34, p-value = 0.0029; A-fragmentation n.s.). Double-strand libraries (n = 10) did not show significant associations. Batches of DNA extraction and sequencing are shown in different colors (genomic A5347–A5350 ss in red, MS1–MS5 ss in green and A8231-A8246 ss exomes in purple). Raw p-values from two-sided Wilcoxon rank tests, comparisons for 5′-ends and molecule length remain significant after correcting for ten tests when alpha = 0.05.

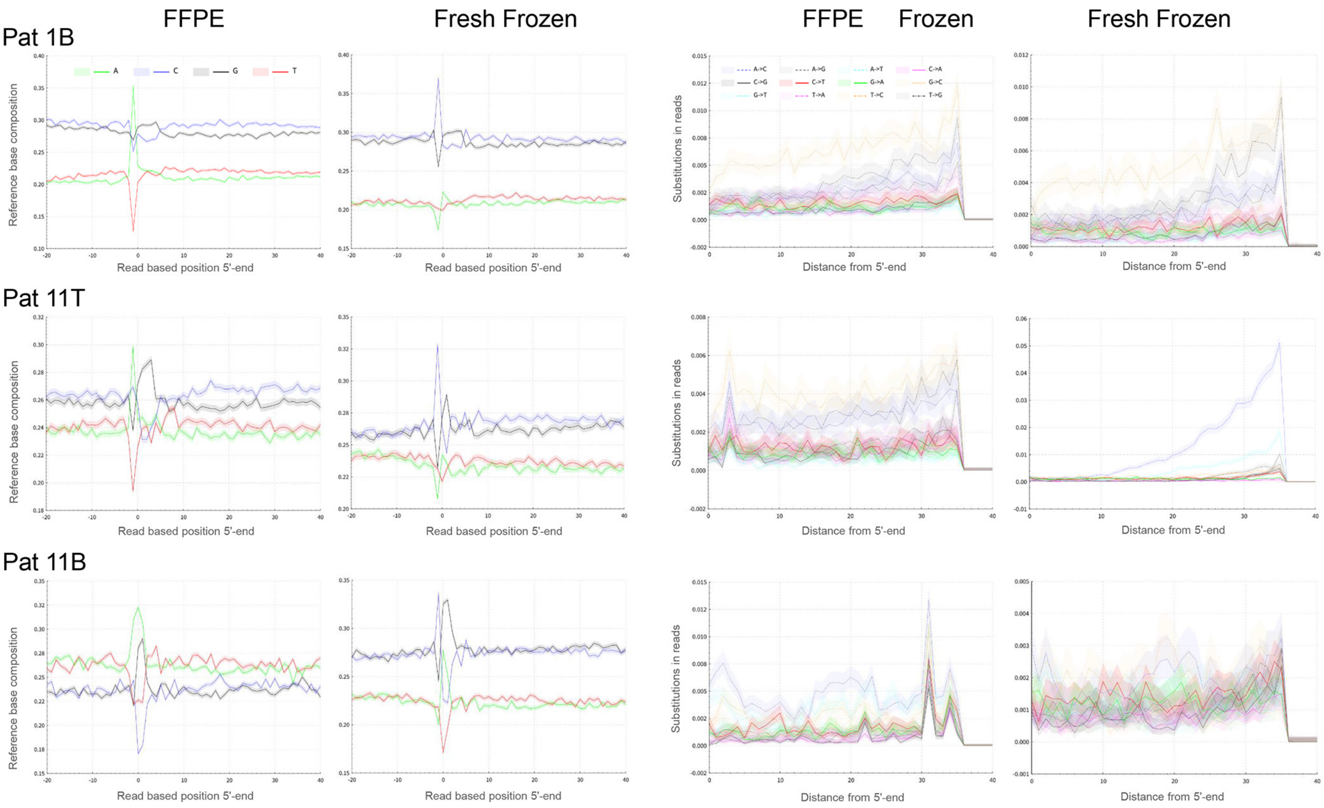

Supplementary Figure S3: Reference base composition and substitution rates in previously published sequencing data (2). Comparison of double-strand DNA library preparations from snap-frozen and FFPE tissues. FFPE DNA shows A-fragmentation, snap-frozen samples show C-fragmentation. FFPE and snap-frozen samples show substitution rates throughout the reads mostly below 2 percent. Pat. Patient. Single end read data, only 5′-end is shown.

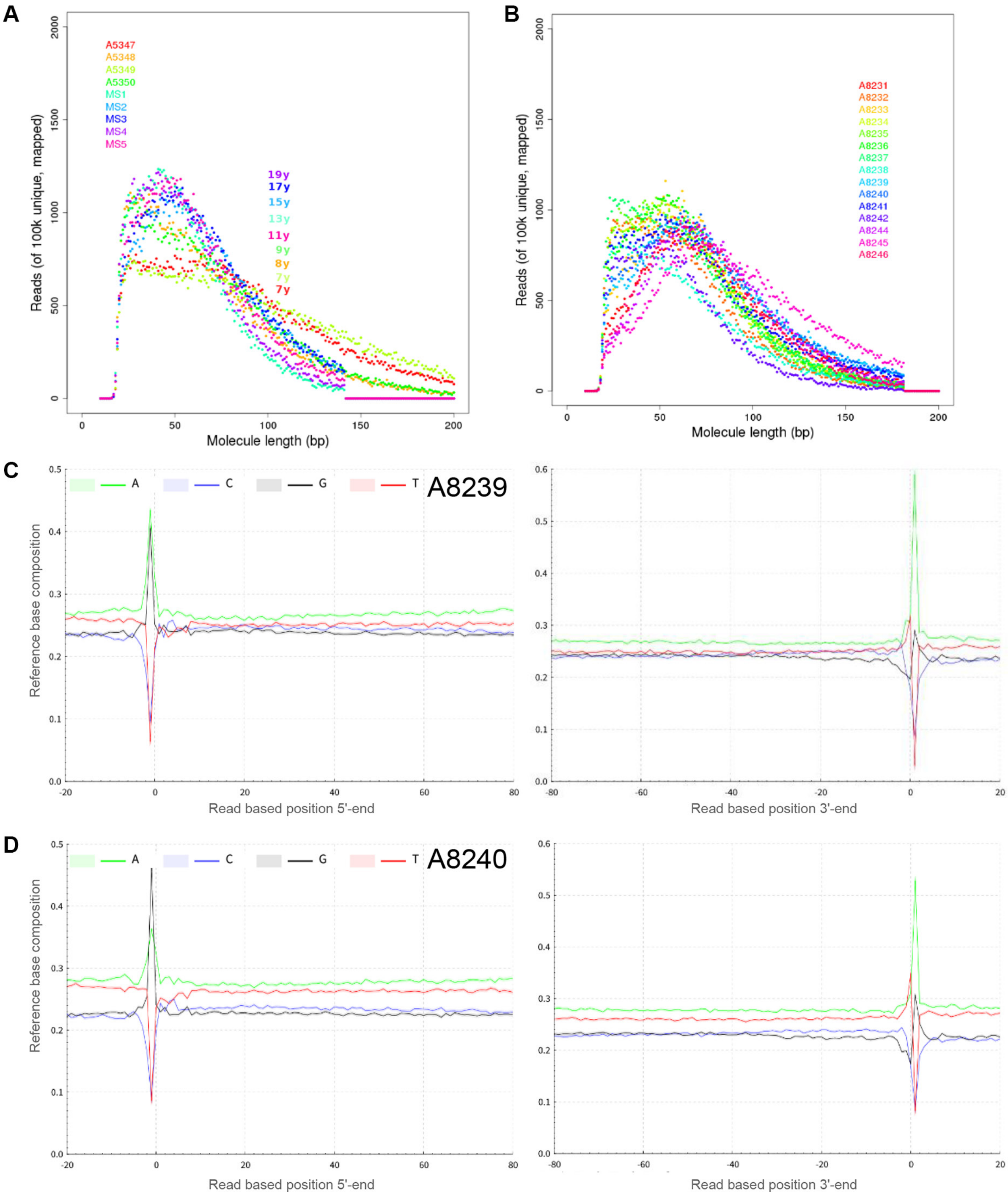

Supplementary Figure S4: Molecule length and reference base composition of FFPE libraries. (**A**) Molecule length in single-strand genomic libraries. Length decreases with sample storage time (given in different color for each sample). As merged reads were analyzed, the plot displays reads with a maximum length of 141 bp (2 × 76 bp reads before merging) for MS1–MS5 libraries. (**B**) Molecule length in single-strand exome libraries. As only merged reads were analyzed, the plot displays reads with a maximum length of 181 bp (2 × 96 bp reads before merging). (**C** and **D**). Reference base composition of FFPE libraries A8239 and A8240, a lung and prostate cancer show elevated adenine and guanine frequencies at the first base outside the sequenced molecule and thus a potential A- and G-fragmentation.

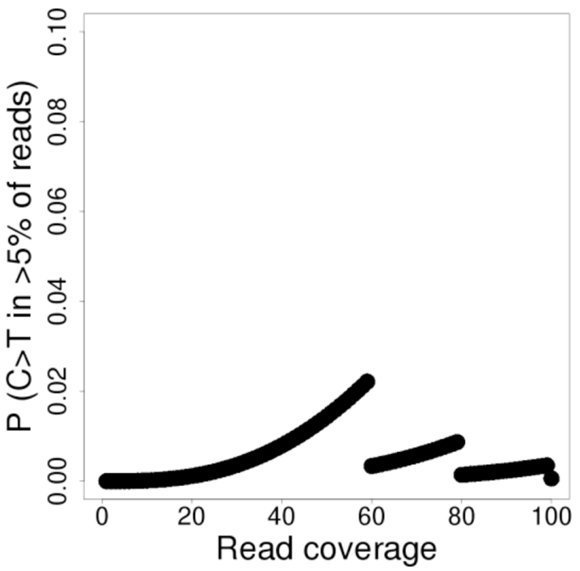

Supplementary Figure S5: Probability of observing false positive variant calls due to C > T damage. Given for the following criteria: minimum of 5% of reads with the variant and at least two reads with the variant required to support variant calls. Modeled using a Poisson distribution for read coverage up to 100x with an expected frequency of C > T in 1% of reads.

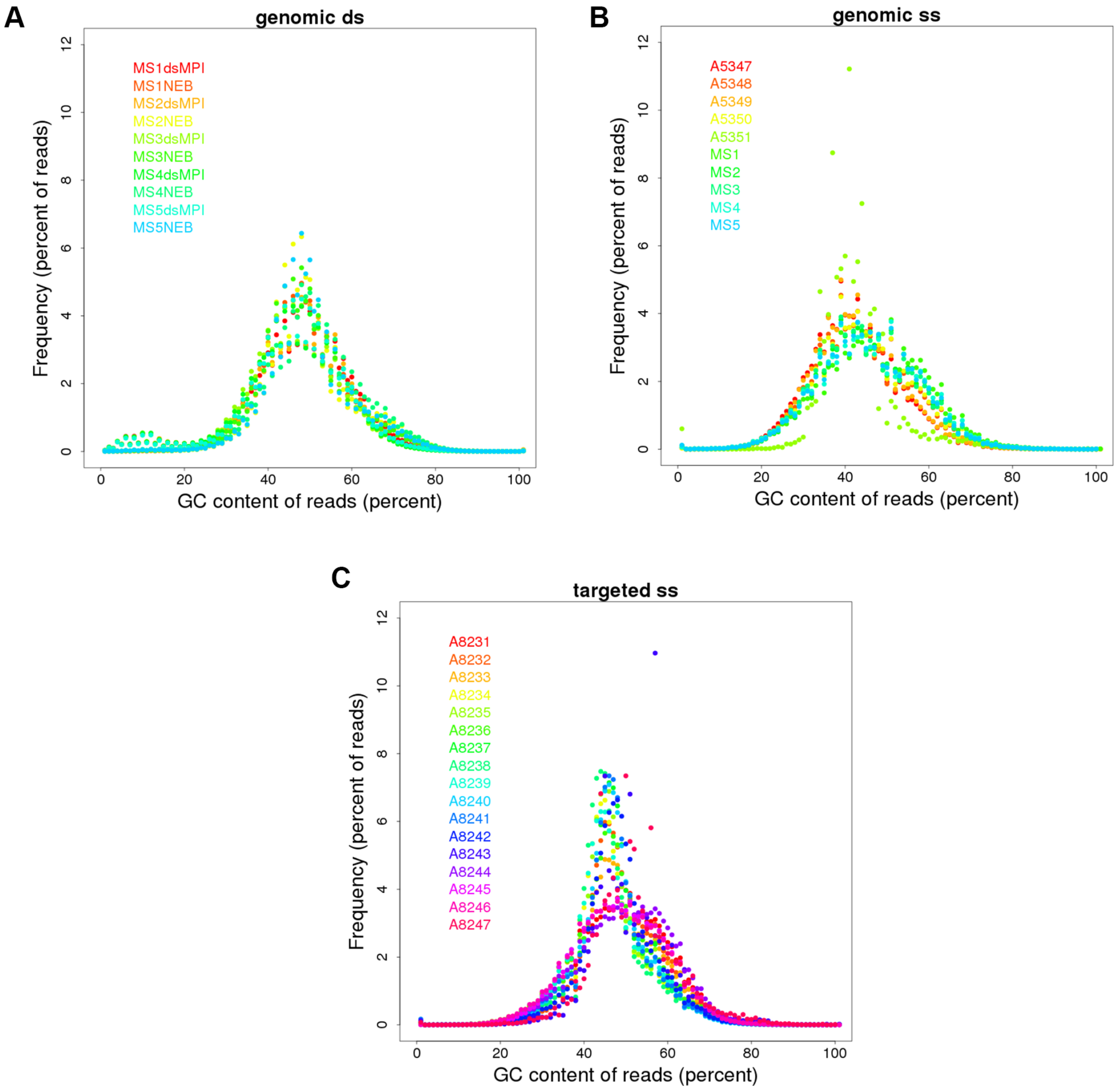

Supplementary Figure S6: GC content of sequencing reads. (**A**) Genomic double-strand libraries and (**B**) single-strand libraries. (**C**) Targeted, exome single-strand libraries.

Supplementary Table S1: Details for sequencing experiments

Sample no.	Cancer	Library preparation	Library ID	Technology
**Initial shotgun sequencing for sequence complexity and DNA damage**				
1	Melanoma	Single-strand	A5347	Ilumina MiSeq130 bp PE
2	Melanoma	Single-strand	A5348	Ilumina MiSeq130 bp PE
3	Melanoma	Single-strand	A5349	Ilumina MiSeq130 bp PE
4	Melanoma	Single-strand	A5350	Ilumina MiSeq130 bp PE
**Shotgun sequencing for comparison of three library preparation methods**				
17	Melanoma	Single-strand<br>Double-strand (MPI)<br>Double-strand (NEB)	MS1<br>MS1dsMPI<br>MS1NEB	Illumina HiSeq2500, 76 bp PE<br>Illumina MiSeq, 150 bp PE<br>Illumina MiSeq, 150 bp PE
18	Melanoma	Single-strand<br>Double-strand (MPI)<br>Double-strand (NEB)	MS2<br>MS2dsMPI<br>MS2NEB	Illumina HiSeq2500, 76 bp PE<br>Illumina MiSeq, 150 bp PE<br>Illumina MiSeq, 150 bp PE
19	Melanoma	Single-strand<br>Double-strand (MPI)<br>Double-strand (NEB)	MS3<br>MS3dsMPI<br>MS3NEB	Illumina HiSeq2500, 76 bp PE<br>Illumina MiSeq, 150 bp PE<br>Illumina MiSeq, 150 bp PE
20	Melanoma	Single-strand<br>Double-strand (MPI)<br>Double-strand (NEB)	MS4<br>MS4dsMPI<br>MS4NEB	Illumina HiSeq2500, 76 bp PE<br>Illumina MiSeq, 150 bp PE<br>Illumina MiSeq, 150 bp PE
21	Melanoma	Single-strand<br>Double-strand (MPI)<br>Double-strand (NEB)	MS5<br>MS5dsMPI<br>MS5NEB	Illumina HiSeq2500, 76 bp PE<br>Illumina MiSeq, 150 bp PE<br>Illumina MiSeq, 150 bp PE
**Exome sequencing for variant calling**				
22	Lung cancer	Single-strand	A8231	Illumina HiSeq, 96 bp PE, rapid mode
23	Breast cancer	Single-strand	A8232	Illumina HiSeq, 96 bp PE, rapid mode
24	Colorectal cancer	Single-strand	A8233	Illumina HiSeq, 96 bp PE, rapid mode
25	Prostate cancer	Single-strand	A8234	Illumina HiSeq, 96 bp PE, rapid mode
26	Colorectal cancer	Single-strand	A8235	Illumina HiSeq, 96 bp PE, rapid mode
27	Lung cancer	Single-strand	A8236	Illumina HiSeq, 96 bp PE, rapid mode
28	Breast cancer	Single-strand	A8237	Illumina HiSeq, 96 bp PE, rapid mode
29	Prostate cancer	Single-strand	A8238	Illumina HiSeq, 96 bp PE, rapid mode
30	Lung cancer	Single-strand	A8239	Illumina HiSeq, 96 bp PE, rapid mode
31	Prostate cancer	Single-strand	A8240	Illumina HiSeq, 96 bp PE, rapid mode
32	Colorectal cancer	Single-strand	A8241	Illumina HiSeq, 96 bp PE, rapid mode
33	Breast cancer	Single-strand	A8242	Illumina HiSeq, 96 bp PE, rapid mode
14	Melanoma	Single-strand	A8244	Illumina HiSeq, 96 bp PE, rapid mode
15	Melanoma	Single-strand	A8245	Illumina HiSeq, 96 bp PE, rapid mode
16	Melanoma	Single-strand	A8246	Illumina HiSeq, 96 bp PE, rapid mode

Initial shotgun sequencing of four single-strand libraries showed sufficient sequence complexity for single-strand libraries (Supplementary Figure S1). The comparison of all three methods was performed on 5 samples. Single-strand library preparation, exome capture and sequencing was then performed on 15 cancer FFPE DNAs to obtain variant calls from scarce FFPE tissue samples.

Supplementary Table S2: Frequencies of A-fragmentation, C > T substitution at molecule ends and molecule length for single-strand and double-strand libraries

Storage time (years)	Library type	Library ID	Batch	A-outside 5′-end	A-outside 3′-end	C > T 5′-end	C > T 3′-end	Median molecule length (bp)
7	ss	A5347	1	0.523	0.634	6.98E-003	1.23E-002	76
8	ss	A5348	1	0.516	0.624	1.95E-002	2.62E-002	58
7	ss	A5349	1	0.468	0.526	6.84E-003	1.34E-002	81
9	ss	A5350	1	0.514	0.61	9.34E-003	1.56E-002	62
13	ss	MS1	2	0.515	0.589	2.18E-002	2.49E-002	51
15	ss	MS2	2	0.487	0.605	2.20E-002	2.46E-002	61
17	ss	MS3	2	0.469	0.56	1.92E-002	2.61E-002	60
19	ss	MS4	2	0.491	0.583	2.46E-002	3.21E-002	52
11	ss	MS5	2	0.531	0.598	1.65E-002	2.54E-002	57
13	ds	MS1dsMPI	3	0.579	0.578	1.38E-002	1.58E-002	76
15	ds	MS2dsMPI	3	0.584	0.583	1.43E-002	1.50E-002	86
17	ds	MS3dsMPI	3	0.57	0.567	1.31E-002	1.34E-002	86
19	ds	MS4dsMPI	3	0.588	0.589	1.49E-002	1.71E-002	79
11	ds	MS5dsMPI	3	0.583	0.584	1.29E-002	1.44E-002	82
13	ds	MS1NEB	4	0.566	0.565	1.37E-002	1.36E-002	88
15	ds	MS2NEB	4	0.528	0.539	6.85E-003	1.68E-002	92
17	ds	MS3NEB	4	0.527	0.531	5.98E-003	1.59E-002	99
19	ds	MS4NEB	4	0.551	0.561	5.92E-003	1.71E-002	87
11	ds	MS5NEB	4	0.564	0.57	5.97E-003	1.42E-002	88
11	ss	A8231	5	0.418	0.524	2.75E-002	3.19E-002	72
11	ss	A8232	5	0.441	0.537	2.28E-002	3.28E-002	57
11	ss	A8233	5	0.379	0.413	2.37E-002	3.21E-002	64
11	ss	A8234	5	0.444	0.562	2.00E-002	2.74E-002	60
9	ss	A8235	5	0.491	0.66	2.96E-002	3.79E-002	59
9	ss	A8236	5	0.502	0.646	2.95E-002	3.65E-002	62
9	ss	A8237	5	0.455	0.576	1.93E-002	2.36E-002	59
9	ss	A8238	5	0.367	0.445	1.21E-002	1.61E-002	57
6	ss	A8239	5	0.434	0.59	7.89E-003	1.55E-002	71
6	ss	A8240	5	0.363	0.528	4.18E-003	1.00E-002	71
6	ss	A8241	5	0.392	0.455	1.50E-002	2.26E-002	65
6	ss	A8242	5	0.498	0.615	2.10E-002	3.33E-002	55
9	ss	A8244	5	0.489	0.556	1.83E-002	1.99E-002	75
4	ss	A8245	5	0.402	0.489	4.86E-003	6.25E-003	86
4	ss	A8246	5	0.54	0.646	1.03E-002	1.57E-002	71
n.a.	ds	Pat11B	6	0.31	n.a.	1.99E-003	n.a.	n.a.
0	ds	Pat11B	6	0.2	n.a.	1.28E-003	n.a.	n.a.
n.a.	ds	Pat11T	6	0.299	n.a.	1.01E-003	n.a.	n.a.
0	ds	Pat11T	6	0.208	n.a.	1.32E-003	n.a.	n.a.
n.a.	ds	Pat1B	6	0.351	n.a.	1.40E-003	n.a.	n.a.
0	ds	Pat1B	6	0.174	n.a.	1.24E-003	n.a.	n.a.

As data from short-term stored samples were single read data, only the 5′-end was ascertained and molecule length could not be ascertained, n.a. (2).

Supplementary Table S3: Average coverage and nonsynonymous coding variant calls reached in exome sequencing

Library ID	Cancer	Mapped reads on genome	Mapped reads on exome	Fraction	Fold enrichment	Unique reads coverage	Variants
A8231	Lung cancer	8880352	3664128	0.41	41.26	4.94	1201
A8232	Breast cancer	8590615	4673607	0.54	54.40	0.33	60
A8233	Colorectal cancer	11548822	5440538	0.47	47.11	2.21	316
A8234	Prostate cancer	9111893	4503396	0.49	49.42	0.63	97
A8235	Colorectal cancer	9302472	4884491	0.53	52.51	0.62	60
A8236	Lung cancer	9510227	4625240	0.49	48.63	0.99	106
A8237	Breast cancer	8905778	4674789	0.52	52.49	0.47	121
A8238	Prostate cancer	8283185	4598067	0.56	55.51	0.23	58
A8239	Lung cancer	8406977	3801180	0.45	45.21	1.57	235
A8240	Prostate cancer	6549725	3043865	0.46	46.47	1.03	118
A8241	Colorectal cancer	7089421	3557527	0.50	50.18	0.59	54
A8242	Breast cancer	7085969	3872693	0.55	54.65	0.26	36
A8243	Negative control	394319	114244	0.29	28.97	0.01	n.d.
A8244	Melanoma	9184487	4079825	0.44	44.42	5.23	1247
A8245	Melanoma	8806918	3742592	0.42	42.50	4.32	1087
A8246	Melanoma	11778127	5246793	0.45	44.55	5.50	1171
A8247	Negative control	295176	117051	0.40	39.65	0.01	n.d.

Enrichment was calculated with genome size of 3.3 Gb and exome size of 33 Mb. See Supplementary Table S6 for all nonsynonymous coding variant calls. N.d. not determined.

Supplementary Table S4: Substitution rates of variants called from exome sequencing of single strand FFPE DNA libraries

library	A > C	A > G	A > T	C > A	C > G	C > T	G > A	G > C	G > T	T > A	T > C	T > G
A8231 lung (n = 1200)	3	10	3.25	5.67	5.5	24.8	21.7	5.17	5.17	2.92	9.83	3.08
A8233 colon (n = 315)	1.9	10.5	0.63	6.35	5.4	23.5	15.6	9.21	2.54	4.76	19.4	0.32
A8239 lung (n = 234)	1.28	12.4	1.71	12.4	3.85	20.9	14.1	5.56	8.12	2.56	14.1	2.99
A8240 prostate (n = 117)	0.85	12	2.56	5.13	5.13	26.5	32.5	3.42	0.85	9.4	1.71	0
A8244 melanoma (n = 1246)	2.97	10.9	1.52	4.49	5.06	27	19.2	5.86	5.14	2.89	12.3	2.73
A8245 melanoma (n = 1086)	2.49	13	1.29	5.71	5.99	20.1	14.7	8.56	6.72	2.58	15.4	3.5
A8246 melanoma (n = 1170)	4.19	12.2	1.54	5.98	5.73	23.8	17	5.04	5.98	2.99	12.8	2.74

Percent of all substitutions.

Supplementary Table S5: GC content of sequenced FFPE DNA libraries

Library type	Library	Percent GC of sequencing reads (median)
Double-strand genomic	MS1dsMPI	48
	MS1NEB	48
	MS2dsMPI	48
	MS2NEB	48
	MS3dsMPI	46
	MS3NEB	48
	MS4dsMPI	46
	MS4NEB	50
	MS5dsMPI	47
	MS5NEB	49
Single-strand genomic	A5347	41
	A5348	43
	A5349	42
	A5350	44
	A5351	41
	MS1	47
	MS2	45
	MS3	44
	MS4	45
	MS5	45
Single-strand targeted exomes	A8231	49
	A8232	48
	A8233	47
	A8234	45
	A8235	46
	A8236	47
	A8237	47
	A8238	45
	A8239	45
	A8240	47
	A8241	47
	A8242	48
	A8243	51
	A8244	51
	A8245	48
	A8246	48
	A8247	51

Supplementary Table S6: Coding mutations called in exome sequencing data from two 10 µm-sections of ~25 square mm per FFPE tissue. Tab names indicates the sequenced libraries. See Supplementary_Table_S6
